# Supplementary material for: The prevalence and correlates of obstructive lung disease among adults aged 45 and above in India: Findings from the longitudinal aging study in India
Source: PLoS One. 2025 Aug 29;20(8):e0327413. doi: 10.1371/journal.pone.0327413 (PMC12396680; doi:10.1371/journal.pone.0327413)
Supplement: S1 Fig — (PDF) [file pone.0327413.s001.pdf]

Fig S1. Flowchart illustrating sample selection processes.

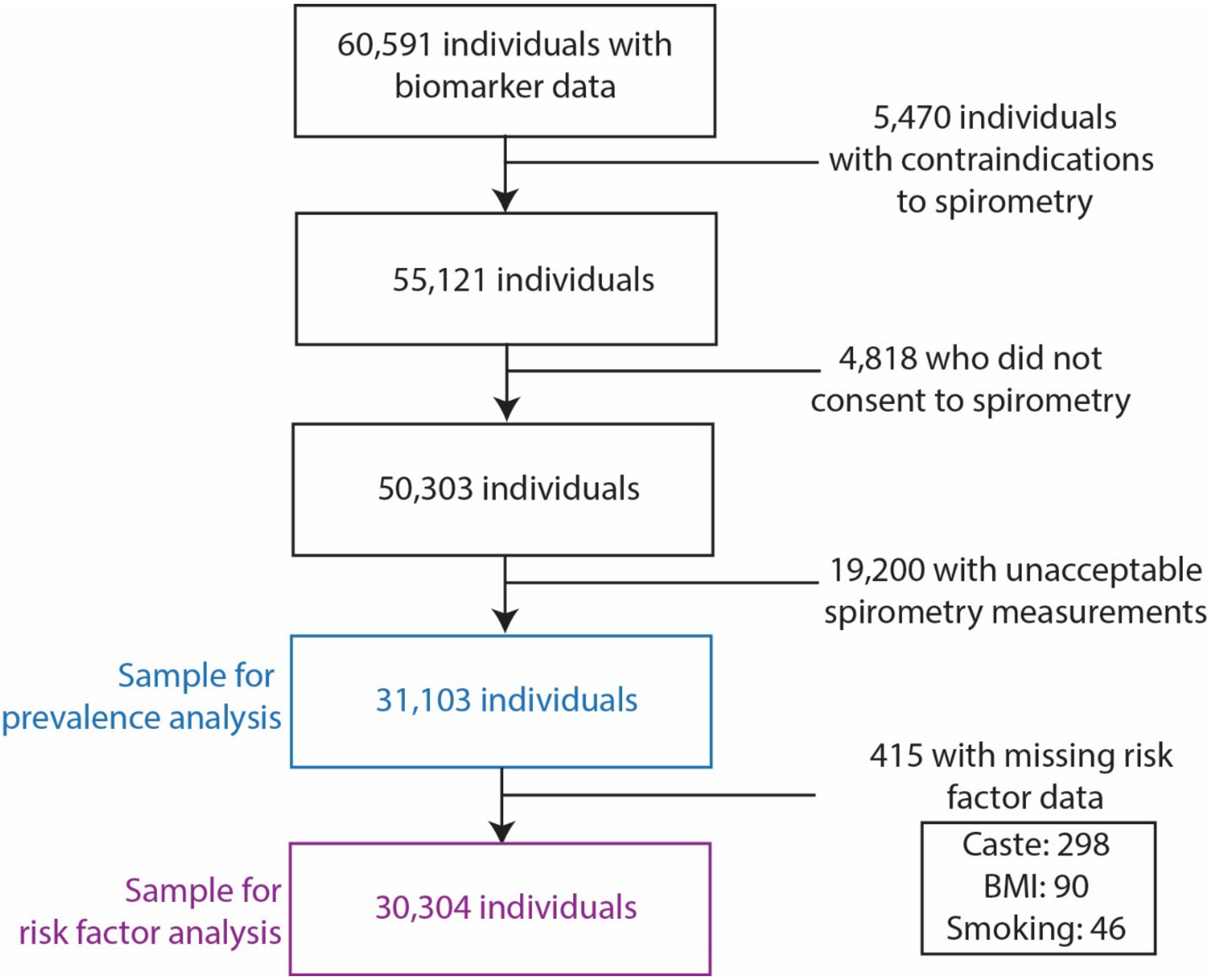

*Contraindications to spirometry include taking tuberculosis medication or current upper respiratory infection, pregnancy, chest or abdominal surgery in the past three months, heart attack or hospitalization for heart problems in the last three months, and eye surgery in the last three months. BMI = body mass index.
