# Supplementary material for: The prevalence and correlates of obstructive lung disease among adults aged 45 and above in India: Findings from the longitudinal aging study in India
Source: PLoS One. 2025 Aug 29;20(8):e0327413. doi: 10.1371/journal.pone.0327413 (PMC12396680; doi:10.1371/journal.pone.0327413)
Supplement: S1 File — (PDF) [file pone.0327413.s002.pdf]

S1 File. Details on other measured variables included in analyses.

- Age: self-reported from survey data
- Gender: self-reported from survey data
- Education: self-reported from survey data, categorized as no school/less than secondary school/secondary school or higher
- Literacy: self-reported from survey data, ability to read or write
- Marital status: self-reported from survey data, married or partnered
- Caste: self-reported from survey data, categorized as no caste or other caste/scheduled tribe/scheduled caste/other backward caste
- Smoking status: self-reported from survey data, categorized as current smoker/former smoker/never smoker; cigarettes, bidis, cigars, hookah, or cheroot were considered in our classifications of smoking status
- Frequency of moderate and vigorous physical activity: self-reported from survey data, categorized as at least once a week/less than once a week
- Use of unclean cooking fuel: self-reported from survey data, unclean fuels include kerosene, charcoal/lignite/coal, crop residue, wood/shrub, dung cake
- Tuberculosis: self-reported from survey data, doctor diagnosis of tuberculosis in the last two years
- Activities of daily living: self-reported from survey data, included dressing, walking across a room, bathing, eating, getting in/out of bed, toileting
- Instrumental activities of daily living: self-reported from survey data, included making phone calls, taking medications, managing money, shopping, preparing meals, getting around, doing housework
- Body mass index: based on measured height and weight, categorized using both World Health Organization (underweight: <18.5 kg/m^2; normal weight: 18.5–24.9 kg/m^2; overweight: 25–29.9 kg/m^2; obese: ≥ 30 kg/m^2) and Indian-specific thresholds (underweight: <18 kg/m^2; normal weight: 18–22.9 kg/m^2; overweight: 23–24.9 kg/m^2; obese: >25 kg/m^2).
