# Supplementary material for: The prevalence and correlates of obstructive lung disease among adults aged 45 and above in India: Findings from the longitudinal aging study in India
Source: PLoS One. 2025 Aug 29;20(8):e0327413. doi: 10.1371/journal.pone.0327413 (PMC12396680; doi:10.1371/journal.pone.0327413)
Supplement: S1 Table — (PDF) [file pone.0327413.s003.pdf]

S1 Table. Distribution of body mass index for Indian-specific and WHO categories

S1 Table. Distribution of body mass index (BMI) variables in the overall sample and stratified by region. WHO refers to World Health Organization.

	**Overall (N = 31,103)**	**North (N = 5,372)**	**Central (N = 2,582)**	**East (N = 7,237)**	**Northeast (N = 6,348)**	**West (N = 3,662)**	**South (N = 5,902)**
BMI category (Indian-specific)							
Normal	16.4 (3,678)	11.8 (411)	24.8 (499)	19.8 (1,319)	17.4 (684)	12.6 (368)	10.7 (397)
Underweight	41.4 (12,899)	36.6 (1,824)	45.8 (1,257)	47.2 (3,373)	51.5 (3,103)	38.2 (1,326)	35.9 (2,016)
Overweight	14.5 (4,914)	16.6 (895)	10.9 (308)	13.8 (1,017)	13.8 (1,064)	15.6 (598)	16.6 (1,032)
Obese	26.5 (9,522)	33.9 (2,226)	17.4 (512)	18.7 (1,509)	17.1 (1,479)	32.9 (1,363)	34.2 (2,433)
Missing	1.3 (90)	1.1 (16)	1.1 (6)	0.5 (19)	0.2 (18)	0.7 (7)	2.6 (24)
BMI category (WHO)							
Normal	20.3 (4,790)	15.0 (545)	30.3 (653)	24.0 (1,634)	22.0 (934)	16.5 (469)	13.5 (555)
Underweight	52.0 (16,701)	50.1 (2,585)	51.2 (1,411)	56.9 (4,075)	60.7 (3,917)	49.9 (1,823)	49.7 (2,890)
Overweight	20.2 (7,177)	24.0 (1,505)	13.5 (406)	15.4 (1,218)	15.0 (1,256)	24.4 (988)	26.2 (1,804)
Obese	6.2 (2,345)	9.9 (721)	3.8 (106)	3.2 (291)	2.1 (223)	8.5 (375)	7.9 (629)
Missing	1.3 (90)	1.1 (16)	1.1 (6)	0.5 (19)	0.2 (18)	0.7 (7)	2.6 (24)
