# Supplementary material for: The prevalence and correlates of obstructive lung disease among adults aged 45 and above in India: Findings from the longitudinal aging study in India
Source: PLoS One. 2025 Aug 29;20(8):e0327413. doi: 10.1371/journal.pone.0327413 (PMC12396680; doi:10.1371/journal.pone.0327413)
Supplement: S2 Table — (PDF) [file pone.0327413.s004.pdf]

S2 Table. Prevalence ratios using Indian-specific and WHO BMI thresholds.

S2 Table. Mean forced expiratory volume in one second/forced vital capacity (FEV_1/FVC), prevalence of obstructive lung disease, and prevalence ratios (PR) for obstructive lung disease by body mass index (BMI) based on Indian-specific and World Health Organization (WHO) thresholds in the Longitudinal Aging Study in India (N=31,103). 95% confidence intervals are shown in parentheses. PRs are derived from Poisson regression models with robust variance. REF is used to denote reference categories. Model 1 adjusts for age and gender. Model 2 additionally adjusts for smoking status.

	Mean FEV_1/FVC	Chronic lung disease prevalence	Chronic lung disease PR (unadjusted)	Chronic lung disease PR (model 1)	Chronic lung disease PR (model 2)
BMI category (Indian-specific)					
Normal	78.6 (78.2–79.0)	15.3 (14.1–16.6)	REF	REF	REF
Underweight	77.1 (76.3–77.9)	22.8 (20.3–25.4)	1.49 (1.33–1.66)	1.35 (1.21–1.51)	1.30 (1.17–1.45)
Overweight	79.7 (79.2–80.2)	11.1 (9.6–12.7)	0.72 (0.63–0.84)	0.76 (0.66–0.88)	0.78 (0.67–0.89)
Obese	80.3 (79.9–80.6)	9.7 (8.7–10.7)	0.63 (0.56–0.71)	0.71 (0.63–0.80)	0.74 (0.66–0.83)
BMI category (WHO)					
Normal	79.0 (78.6–79.4)	13.8 (12.8–14.9)	REF	REF	REF
Underweight	77.3 (76.5–78.0)	22.2 (19.9–24.6)	1.61 (1.45–1.78)	1.46 (1.32–1.62)	1.39 (1.26–1.54)
Overweight	80.1 (79.7–80.4)	10.0 (9.0–11.1)	0.73 (0.64–0.82)	0.78 (0.69–0.89)	0.81 (0.71–0.91)
Obese	80.9 (80.3–81.5)	8.6 (6.9–10.7)	0.62 (0.50–0.78)	0.76 (0.60–0.96)	0.80 (0.63–1.00)
