# Supplementary material for: The prevalence and correlates of obstructive lung disease among adults aged 45 and above in India: Findings from the longitudinal aging study in India
Source: PLoS One. 2025 Aug 29;20(8):e0327413. doi: 10.1371/journal.pone.0327413 (PMC12396680; doi:10.1371/journal.pone.0327413)
Supplement: S2 File — (PDF) [file pone.0327413.s005.pdf]

S2 File. Details on covariates included in inverse probability weights.

Covariates included in inverse probability weight models: State, age, gender, education, literacy, marital status, rural/urban residence, caste, body mass index category, self-reported moderate and vigorous physical activity, the sum of activities of daily living and instrumental activities of daily living limitations, and respiratory symptoms (coughing, dizziness, shortness of breath, wheezing). We also considered interactions among gender and age, education, and body mass index category.
