# Supplementary material for: The prevalence and correlates of obstructive lung disease among adults aged 45 and above in India: Findings from the longitudinal aging study in India
Source: PLoS One. 2025 Aug 29;20(8):e0327413. doi: 10.1371/journal.pone.0327413 (PMC12396680; doi:10.1371/journal.pone.0327413)

Fig S2. Standardized mean differences for inverse probability weights

Fig S2. Weighted and unweighted standardized mean differences for important characteristics comparing the full Longitudinal Aging Study in India sample to the subsample with available and acceptable spirometry data. Vertical lines illustrate the region for which differences are considered acceptable (±0.1). We evaluated standardized mean differences for continuous variables and crude differences for binary or categorical variables. BMI refers to body mass index, ADLs to activities of daily living, and IADLs to instrumental activities of daily living.

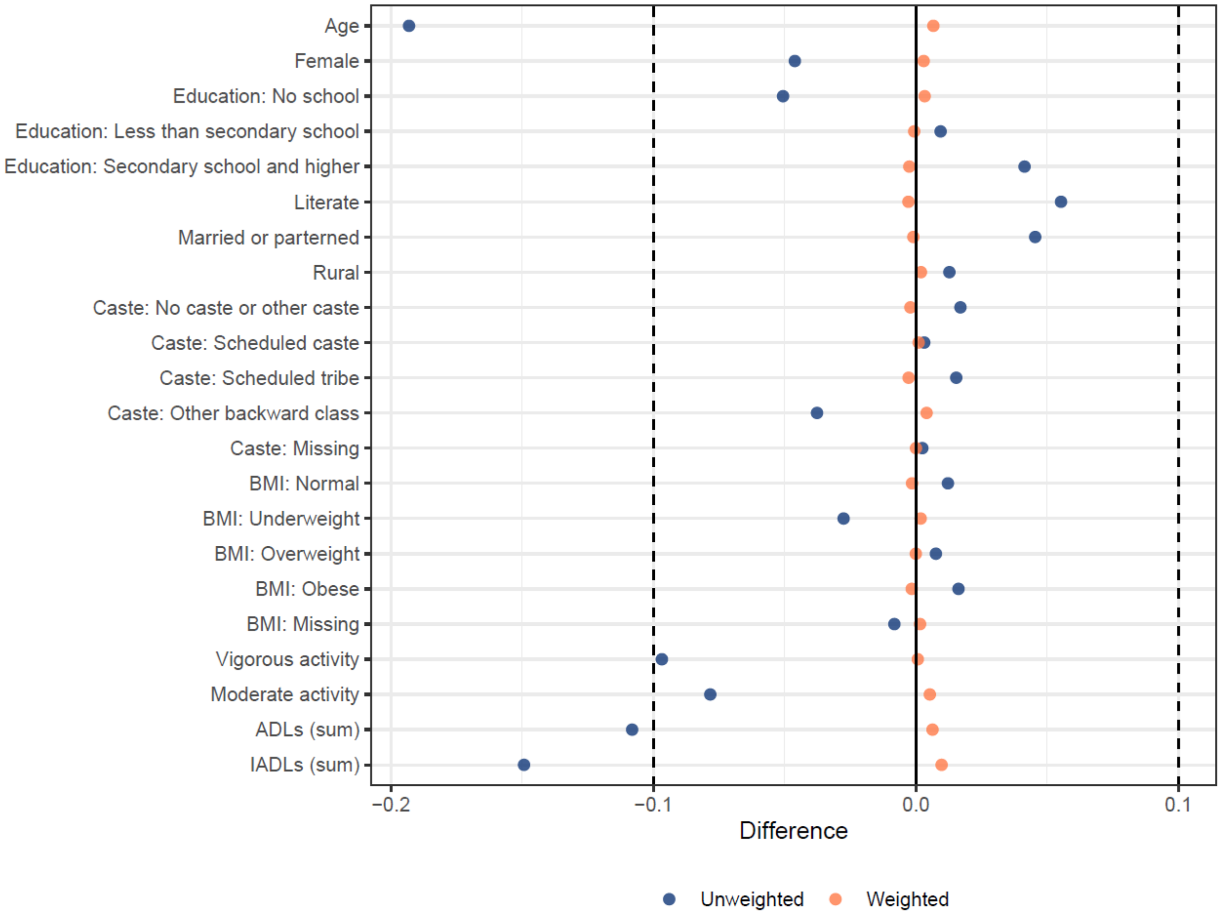

Supplement: S2 Fig — (PDF) [file pone.0327413.s006.pdf]
