# Supplementary figures and images for: The prevalence and correlates of obstructive lung disease among adults aged 45 and above in India: Findings from the longitudinal aging study in India
Source: PLoS One. 2025 Aug 29;20(8):e0327413. doi: 10.1371/journal.pone.0327413 (PMC12396680; doi:10.1371/journal.pone.0327413)

**Fig S3.** Map depicting the categorization of regions within India.

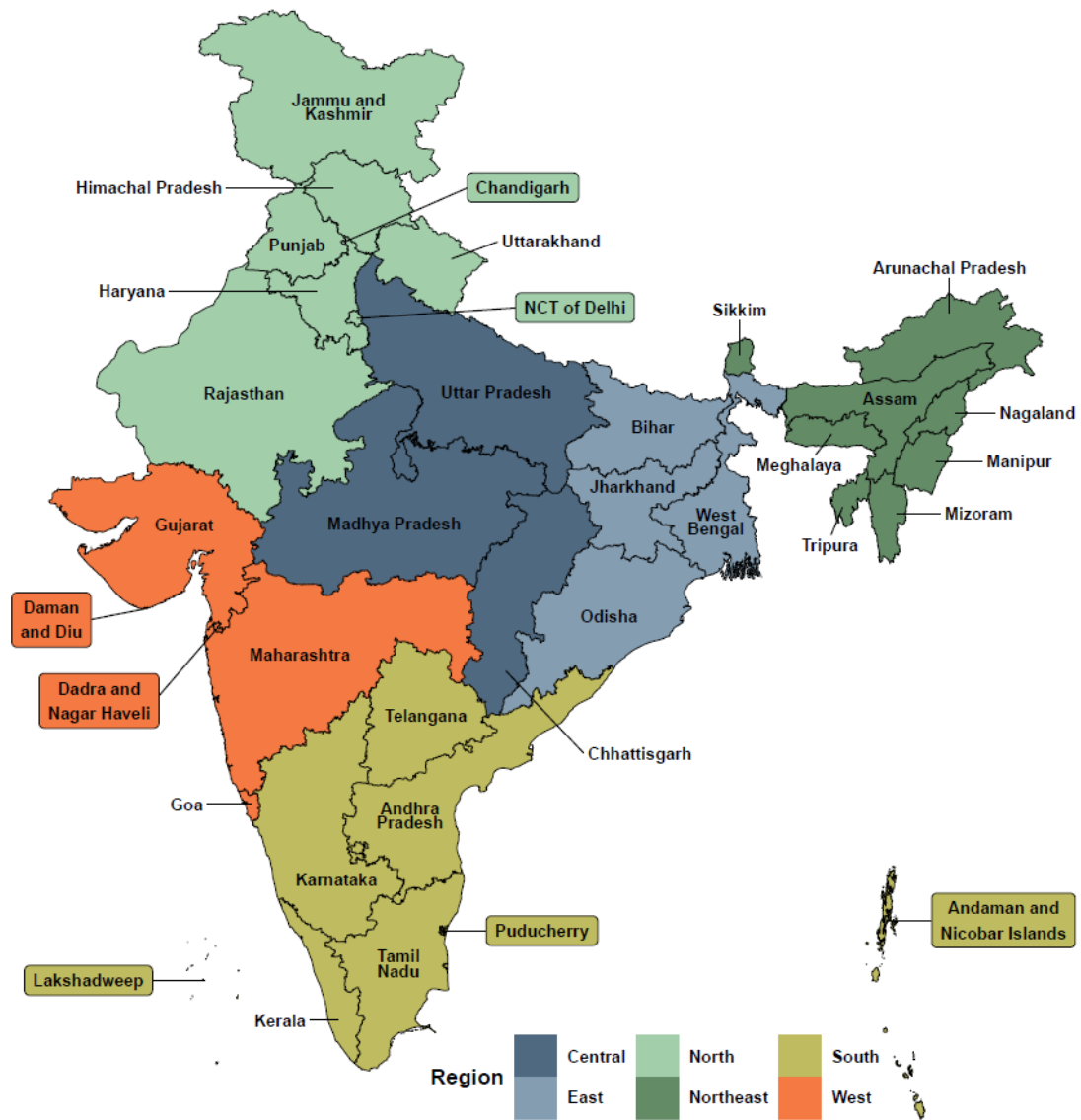

Supplement: S3 Fig — (PDF) [file pone.0327413.s007.pdf]
