# Supplementary material for: The prevalence and correlates of obstructive lung disease among adults aged 45 and above in India: Findings from the longitudinal aging study in India
Source: PLoS One. 2025 Aug 29;20(8):e0327413. doi: 10.1371/journal.pone.0327413 (PMC12396680; doi:10.1371/journal.pone.0327413)
Supplement: S3 Table — (PDF) [file pone.0327413.s008.pdf]

S3 Table. Prevalence by geographic region

S3 Table. Mean forced expiratory volume in one second/forced vital capacity (FEV_1/FVC) and the prevalence and severity of obstructive lung disease nationally and by geographic region among older adults in the Longitudinal Aging Study in India (N=31,103). 95% confidence intervals are shown in parentheses. Means and prevalences are weighted to account for unequal sampling probabilities and selection into the spirometry sample.

	**FEV_1/FVC**	**Overall prevalence**	**Mild**	**Moderate**	**Severe or very severe**
Overall	79.0 (78.6–79.4)	14.4 (13.4–15.4)	2.2 (2.0–2.6)	8.2 (7.6–8.9)	3.9 (3.4–4.4)
North	79.3 (78.3–80.3)	17.2 (15.0–19.6)	3.8 (2.9–5.1)	8.8 (7.4–10.5)	4.5 (3.5–5.8)
Central	79.3 (78.4–80.3)	15.9 (13.7–18.3)	2.4 (1.7–3.4)	9.0 (7.3–10.9)	4.5 (3.7–5.5)
East	79.0 (78.4–79.6)	11.5 (10.0–13.2)	2.0 (1.6–2.6)	6.8 (5.8–8.1)	2.6 (2.0–3.4)
Northeast	79.2 (78.3–80.1)	10.8 (8.2–14.0)	1.4 (1.0–1.9)	7.2 (5.5–9.4)	2.2 (1.3–3.6)
West	77.6 (76.7–78.4)	16.2 (13.8–18.9)	2.1 (1.6–2.7)	9.4 (7.5–11.6)	4.8 (3.5–6.4)
South	79.4 (78.7–80.2)	13.3 (11.2–15.7)	1.7 (1.1–2.5)	7.9 (6.8–9.3)	3.7 (2.6–5.2)
