# Supplementary material for: The prevalence and correlates of obstructive lung disease among adults aged 45 and above in India: Findings from the longitudinal aging study in India
Source: PLoS One. 2025 Aug 29;20(8):e0327413. doi: 10.1371/journal.pone.0327413 (PMC12396680; doi:10.1371/journal.pone.0327413)
Supplement: S4 Table — (PDF) [file pone.0327413.s009.pdf]

S4 Table. Prevalence by age and gender

S4 Table. Prevalence of mild, moderate, and severe or very severe obstructive lung disease among older adults in the Longitudinal Aging Study in India (N=31,103) by gender and age group. 95% confidence intervals are shown in parentheses. Prevalence estimates are weighted to account for unequal sampling probabilities and selection into the spirometry sample.

Severity	Age group	Men	Women
Overall	45–54	0.106 (0.094–0.119)	0.079 (0.068–0.092)
Overall	55–64	0.178 (0.160–0.199)	0.108 (0.096–0.122)
Overall	65–74	0.251 (0.224–0.281)	0.131 (0.111–0.153)
Overall	75+	0.322 (0.273–0.374)	0.181 (0.125–0.254)
Mild	45–54	0.012 (0.008–0.016)	0.014 (0.010–0.020)
Mild	55–64	0.020 (0.015–0.026)	0.019 (0.014–0.026)
Mild	65–74	0.036 (0.028–0.045)	0.028 (0.019–0.040)
Mild	75+	0.044 (0.029–0.068)	0.063 (0.031–0.122)
Moderate	45–54	0.066 (0.058–0.076)	0.046 (0.038–0.056)
Moderate	55–64	0.111 (0.097–0.127)	0.064 (0.055–0.074)
Moderate	65–74	0.146 (0.125–0.170)	0.073 (0.058–0.091)
Moderate	75+	0.170 (0.134–0.213)	0.051 (0.030–0.087)
Severe or very severe	45–54	0.028 (0.022–0.034)	0.019 (0.013–0.026)
Severe or very severe	55–64	0.048 (0.037–0.060)	0.026 (0.019–0.034)
Severe or very severe	65–74	0.070 (0.057–0.086)	0.030 (0.021–0.043)
Severe or very severe	75+	0.108 (0.076–0.150)	0.067 (0.034–0.128)
