# Supplementary material for: The prevalence and correlates of obstructive lung disease among adults aged 45 and above in India: Findings from the longitudinal aging study in India
Source: PLoS One. 2025 Aug 29;20(8):e0327413. doi: 10.1371/journal.pone.0327413 (PMC12396680; doi:10.1371/journal.pone.0327413)
Supplement: S5 Table — (PDF) [file pone.0327413.s010.pdf]

S5 Table. Objective prevalence, self-reported prevalence, and disease awareness by region

S5 Table. Weighted prevalence of obstructive lung disease, prevalence of self-reported lung disease, and disease awareness among middle-aged and older adults in the Longitudinal Aging Study in India (N=31,103) by region. 95% confidence intervals are shown in parentheses.

	Prevalence	Self-reported prevalence	Awareness
North	0.17 (0.15–0.20)	0.05 (0.04–0.06)	0.10 (0.07–0.13)
Central	0.16 (0.14–0.18)	0.05 (0.03–0.06)	0.11 (0.07–0.17)
East	0.11 (0.10–0.13)	0.04 (0.04–0.05)	0.14 (0.10–0.18)
Northeast	0.11 (0.08–0.14)	0.02 (0.02–0.03)	0.07 (0.04–0.13)
West	0.16 (0.14–0.19)	0.05 (0.04–0.07)	0.14 (0.11–0.19)
South	0.13 (0.11–0.16)	0.06 (0.04–0.07)	0.10 (0.07–0.16)
