# Supplementary material for: The prevalence and correlates of obstructive lung disease among adults aged 45 and above in India: Findings from the longitudinal aging study in India
Source: PLoS One. 2025 Aug 29;20(8):e0327413. doi: 10.1371/journal.pone.0327413 (PMC12396680; doi:10.1371/journal.pone.0327413)

Fig S4. Maps showing objective prevalence, self-reported prevalence, and disease awareness by region

Fig S4. Maps showing the weighted prevalence of obstructive lung disease [A], prevalence of self-reported lung disease [B], and disease awareness [C] among middle-aged and older adults in the Longitudinal Aging Study in India (N=31,103) by region.

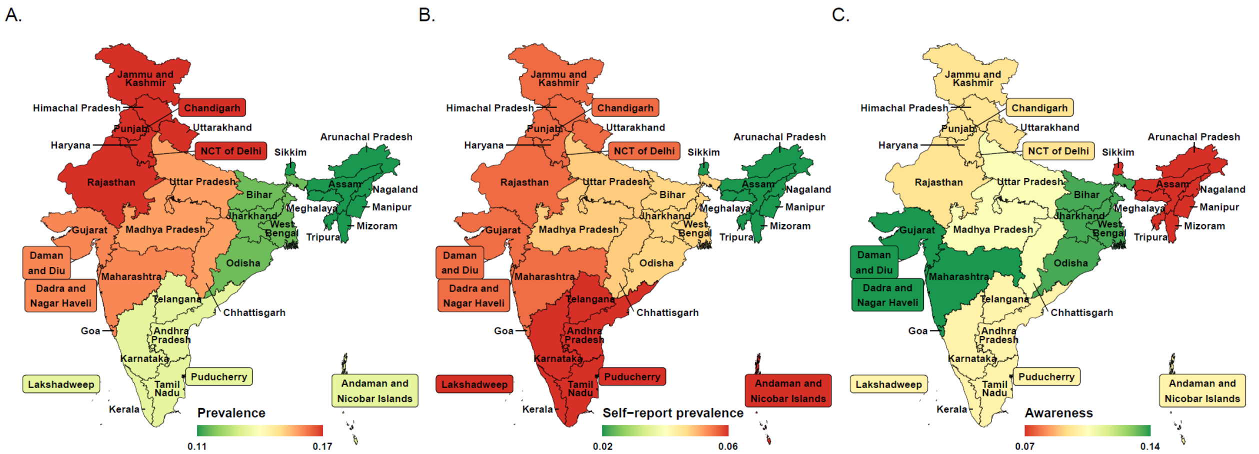

Supplement: S4 Fig — (PDF) [file pone.0327413.s011.pdf]
