# Supplementary material for: The prevalence and correlates of obstructive lung disease among adults aged 45 and above in India: Findings from the longitudinal aging study in India
Source: PLoS One. 2025 Aug 29;20(8):e0327413. doi: 10.1371/journal.pone.0327413 (PMC12396680; doi:10.1371/journal.pone.0327413)
Supplement: S6 Table — (PDF) [file pone.0327413.s012.pdf]

S6 Table. Prevalence comparisons between regions

S6 Table. Prevalence ratios for obstructive lung disease by region among middle-aged and older adults in the Longitudinal Aging Study in India (N=31,103). 95% confidence intervals are shown in parentheses. Prevalence ratios are derived from Poisson regression models with robust variance. REF is used to denote the reference category. Models shown are unadjusted; adjusted for age and gender; adjusted for age, gender, and smoking status; and adjusted for age, gender, and all included risks.

Region	Unadjusted	Adjusted for age and gender	Add smoking status	Add all other risks
Central	1.46 (1.09–1.96)	1.36 (1.00–1.84)	1.35 (1.00–1.82)	1.22 (0.91 - 1.64)
East	1.05 (0.79–1.41)	1.02 (0.76–1.37)	1.03 (0.77–1.38)	0.96 (0.71 - 1.29)
North	1.58 (1.18–2.12)	1.52 (1.13–2.05)	1.43 (1.07–1.91)	1.45 (1.08 - 1.95)
Northeast	REF	REF	REF	REF
South	1.24 (0.91–1.68)	1.21 (0.89–1.65)	1.22 (0.90–1.65)	1.23 (0.90 - 1.68)
West	1.51 (1.10–2.06)	1.47 (1.07–2.00)	1.52 (1.13–2.05)	1.52 (1.13 - 2.05)
